# Early release from prison in time of COVID-19: Determinants of unfavourable decisions towards Black prisoners

**DOI:** 10.1371/journal.pone.0252319

**Published:** 2021-05-27

**Authors:** Mariana P. Miranda, Rui Costa-Lopes, Gonçalo Freitas, Catarina L. Carvalho

**Affiliations:** 1 Institute of Social Sciences, University of Lisbon, Lisbon, Portugal; 2 Faculty of Psychology and Education Science, University of Porto, Porto, Portugal; University of Connecticut, UNITED STATES

## Abstract

On the onset of the COVID-19 pandemic, the overcrowding in prisons led to efforts to decarcerate in order to prevent and control outbreaks in prisons. This study analyses how public support for such exceptional measures are determined by cognitive and ideological factors known to create and maintain racial biases in the criminal system. Participants were asked to express their level of agreement with the early-release of hypothetical prisoners. Results showed participants to be less favourable to the early-release of Black compared to White prisoners, when they had committed a stereotypically Black crime. As expected, the congruency between the crime stereotypicality and the colour of the prisoner’s skin did not emerge for White prisoners. Moreover, the difference between the agreement with the release of the Black vs. the White prisoner when both committed a stereotypically Black crime was higher as the level of endorsement of Meritocracy increased. Contrastingly, Anti-egalitarianism only predicted an overall disagreement with prisoners’ early-release. This paper highlights the cumulative explanation by different levels of analysis of this current problem and implications for the development of the public opinion on penal subjects.

## Introduction

Since 2000 the number of imprisoned people worldwide has increased 24% to a total of 10.74 million people [1]. U.S. prisons alone account for 2.2 million incarcerated people [2]. In the European Union, the context of this research, most recent data shows the existence of 495.000 prisoners [3].

In early 2020, as COVID-19 evolved into a full scale outbreak, prisons were already perceived as potential epicenters for infectious diseases and were, as such, considered part of the pandemic public health response plan [4]. Indeed, despite a significant variation in prison system conditions between countries, a significant number of countries face concerning levels of overcrowding and there is an overall scarcity of medical services [5].

Concurrently, scholars [6] and international organizations argued in favour of early decarceration. In May 2020, a joint statement from the United Nations Office on Drugs and Crime (UNODC), the World Health Organization, United Nations Human Rights Council and the Joint United Nations Programme on HIV/AIDS urged political leaders to reduce overcrowding in prisons and other confined settings in order to prevent and control COVID-19 outbreaks [7]. In particular, the statement appealed for release mechanisms for prisoners both at particular risk of COVID-19 and sentenced for minor, non-violent, offenses.

The latest European Prison Observatory report on June, 2020 [8], detailed different efforts to address these recommendations, including evidence of early release of prisoners due to COVID-19 in France, the U.K. and Portugal. However, these decisions did not emerge without opposing views of citizens and politicians, leading to public debate in mainstream media outlets [9]. This paper focuses precisely on the development of supporting or opposing positions to these political measures by the general public.

Furthermore, given that the COVID-19 pandemic overlaps and unevenly impacts an array of chronical issues in the penal system, in this paper, we zoom in on the discrimination of low-status group members, in particular Black prisoners.

### Discrimination of racial minorities in the criminal system

The ratios of minority-incarcerated population as a function of the overall total of the group population has shown a systematic disadvantage for foreigners and minority groups [10, 11]. Considering different scenarios that take into account variables that capture the social structure of the groups, the likelihood of a migrant being in prison is greater than for a national citizen in every model [12].

Research on decision-making in the legal context shows that other-race low-status group members are targeted with overall more unfavourable decisions in the four main stages of the criminal path [13]. The differences start with racial disparities in arrest rates after police encounters [14], and prosecutor’s charging decisions [15], continue to the trial phase with evidence from mock trials [16, 17], and end with the sentencing phase [17].

Moreover, this overrepresentation seems to be self-feeding. Hetey and Eberhardt [18] experimentally isolated the outcomes of the perception of overrepresentation of racial minorities. The authors found that the more citizens perceive that the majority of the incarcerated population is Black the less responsive they are to actions that tackle those same disparities (e.g., signing a petition to cancel the ethnic profiling—stop-and-frisk program).

The early release of prisoners due to the COVID-19 pandemic allows us to turn the spotlight to a posterior stage: the clemency. The question emerges of whether racial disparities described in the four stages of the criminal path resurface in this add-on stage, involving the decision to early release prisoners due to COVID-19. We begin this discussion by surveying the factors already known to be involved in more unfavourable decisions towards low-status group members in the legal context.

#### Cognitive factors

The formation and maintenance of intergroup relations depend on cognitive mechanisms that guide information processing, such as stereotypes [19]. Stereotypes are shared beliefs about the characteristics of social groups and their members [20].

Research on stereotypes has shown the existence of consequences for the Black group members in several socio-cognitive processes that relate to the disparities described above. It impacts social perception, with the activation of stereotypic associates of Blacks leading subjects to interpret an ambiguous behaviour performed by a race-unspecified target person to be more hostile [21]. This effect was extended to attentional biases, showing that the activation of the concept of crime guides attention towards Black faces and that this is a bidirectional process [22]. As to memory processes, research [23] has shown that people use stereotypes to organize presented evidence in a complex judgment of guilt. As such, they recall more negative information about low-status groups.

Analysing the effects of stereotypes on behaviour, we can acknowledge that these are widespread through the legal and criminal systems. Research on police encounters has studied simulated decisions to shoot or not to shoot. Results show that both community samples and police officers have strong racial bias in the time they take to make a decision, though police training blocked more frequent incorrect shooting decisions toward Black targets [24]. This effect has been partially attributed to stereotypes, in the sense that stereotype-congruent decisions are facilitated, hence faster. A similar effect is the fact that people are quicker to distinguish blurred images of weapons than blurred images of tools when they have been primed before with pictures of Black faces, comparing with White faces [25].

Stereotyping might also be observed in criminal-sentencing decisions. The display of Afrocentric features (dark skin and hair, broad noses, thick lips) predicts the probability of a death sentence verdict for Black defendants. This effect happened only when the victims were White [26]. The negative effect of the stereotypicality of traits was also described in the sentences of inmates. Controlling for the criminal records, inmates with more Afrocentric features received harsher sentences [27].

Furthermore, concerning the studies on mock juror decision-making, a meta-analysis [17] revealed a small but significant racial bias in the sentencing phase, with White participants giving Black defendants longer sentences than to White defendants. In tune, a second meta-analysis [16] reported the existence of a small but significant racial bias, where defendants from a racial outgroup are generally targeted with more negative verdict and sentencing decisions, especially when White jurors evaluate Black defendants. We highlight a third meta-analysis [28] as it explored the moderators that can shed light on the inconsistencies in the racial bias in sentencing. They indeed found a non-significant main effect of racial bias on either trial judgments or sentences in their meta-analysis of 29 studies. However, they observed an effect of the defendant race on sentences qualified by the type of crime, meaning that the recommended sentences were harsher for Blacks than for Whites in cases of negligent homicide, and for Whites than for Blacks in crimes of embezzlement/fraud. It suggests that people have a tendency to associate particular social groups with certain crimes, the so-called crime stereotypicality [29] and that this cognitive element can predict more unfavourable decisions towards Black people in the criminal justice system. Experimental studies with lay participants [30] have isolated this race-crime congruency effect showing that when associated to a stereotypically Black crime, Black defendants are targeted with harsher sentences.

#### Ideological factors

According to social dominance theory, all social systems tend to be organized based on hierarchies, and rely on ideologies, that either promote or attenuate intergroup hierarchies (hierarchy-enhancing vs. hierarchy-attenuating legitimizing myths) [31]. The extent to which these ideologies are accepted by individuals is represented by a social dominance orientation (SDO), reflecting individuals’ desire to establish and maintain hierarchical intergroup relations [31]. The criminal justice system is regarded as one of the most hierarchy-enhancing systems, and not surprisingly, professionals with dominant positions within this system (e.g. police) display higher levels of SDO than the people outside the system [32].

The same type of reasoning applies to lay people, as SDO is associated to more consensus and normalization of inequality, through higher support for discrimination [33], as well as less support for progressive social policies [34]. Drawing a direct parallel with the context of our research, the endorsement of this hierarchy-enhancing ideology predicts higher support for longer prison sentences and other law and order policies [35]. An explanation of the process is that SDO feeds the belief in various forms of criminal deterrence and the moral imperative of criminal retribution [36] and through them justifies support for harsh outcomes [37].

The increased value of egalitarianism observed from the second half of the 20^th^ century [38], made hierarchy-enhancing myths ever more conflicting with societal standards. To this extent, some studies on hierarchy-enhancing myths exclude participants that score higher in social desirability [39]. The same way racism adapted to these new norms [40, 41], so did the prevalent hierarchy-enhancing ideologies. Here, we will focus on one of these: Meritocracy.

Meritocracy is widely accepted [42] and often defended as first defined: a justice principle, through which everyone’s own outcomes are contingent on one’s inputs [43, 44]. Nevertheless, there is a paradox within Meritocracy [45] that is seen as deriving from an equity principle but has been associated to the underestimation of inherent inequalities [46] and to their justification [47]. A systematic review [48] has shown that its endorsement negatively affects low-status group members. These results have been described in important fields, such as in organizations [49], schools [50] and reparation policies [51], but only tangentially in decisions within the criminal justice system [52, 53]. We argue that the results are tangential as these studies focused on the Protestant Work Ethic [54], a norm that shares the dimension of effort-based success with Meritocracy but it is seen only as a component of it [48] and because it does not differentiate decisions towards racialized groups.

### The present research

The present research focuses on the interplay of cognitive and ideological aspects that are known to be associated to more unfavourable decisions towards low-status within the criminal justice system. In particular, the study presented here analyses the role of stereotypes and hierarchy-enhancing ideologies such as SDO and Meritocracy in the support for a very current public policy: the early release of prisoners due to the COVID-19 pandemic.

The first hypothesis examined the effect of the congruency between skin colour and crime stereotypicality in a new stage of the criminal path: the decision to early-release prisoners. In line with literature in the sentencing phase [30] that we expect to translate to these clemency decisions, we predict that Black prisoners will be discriminated in COVID-19-related early release from prison when there is a congruency between the crime and the prisoner. More precisely, we expect participants to disagree more with early release of Black rather than White prisoners when the crime committed is stereotypically Black. Contrastingly, we do not predict the same effect in White prisoners comparing to Black prisoners when they commit a stereotypically White crime.

The second hypothesis directly investigated the integration of both cognitive and ideological aspects. There have been efforts in this sense. For instance, conservatives perceive affirmative action as merit-violating (at least in part) because they see beneficiaries as less deserving [55]. In the same sense, we expect hierarchy-enhancing ideologies to produce negative decisions towards prisoners in part because crime stereotypicality congruency informs on the stability of the factors leading to the crime [56].

Based solely on the literature described above, we expect that the degree to which participants agree with the early release of Black prisoners will be negatively correlated to the endorsement of legitimizing beliefs, in particular for those who committed a stereotypically Black crime. As such we have a prediction for both main effects from the hierarchy enhancing ideologies and the interaction between the hierarchy enhancing ideologies and the cognitive factor.

This hypothesis was pre-registered and is based on an ethnocentric motivation, with an effect of hierarchy-enhancing ideologies within the intergroup discrimination. However, an alternative predicting has to be mentioned, because of the particular context of the intergroup comparison, with White participants expressing support or opposition to the release of targets, both White and Black, who are prisoners, considered a low-status group and thus, per se, a very stigmatized group [57]. As such, we could also sustain an hypothesis favouring a generalized prejudice effect (towards prisoners), in which prisoners are discriminated against by people endorsing hierarchy-enhancing ideologies, regardless of being Black or White.

To test these hypotheses, we will focus on SDO and Meritocracy. SDO is composed by two related but distinct subdimensions: support for group-based dominance hierarchies and general opposition to equality [39, 58]. Separate analyses of these two dimensions result in more refined predictions, with group dominance being indicated for the analysis of conflict and extreme attitudes and Anti-egalitarianism being advocated as more suitable for the study of social policies related to relatively subtle hierarchy-enhancing legitimizing ideologies [58]. We could argue that the criminal justice system outcome discrepancies are extreme. However, we will highlight the analysis of the inequality-driven motivation for outcomes for prisoners. First because earlier papers conducted by SDO theorists made salient the dominance motive. Second because the context of the decision is supported by a subtle need-based argument (the need to reduce overcrowding due to the COVID-19 pandemic). And finally, because it makes a more stringent comparison with the content of the second hierarchy-enhancing ideology tested here.

Regarding Meritocracy, the second hierarchy-enhancing ideology, an important distinction has previously been made between defending the merit principle (prescriptive Meritocracy) and the belief that the system works based on merit (descriptive Meritocracy). Only the latter is related to other hierarchy-legitimizing ideologies and a predictor of prejudice [59] and, as such, we will focus on the latter [60].

The extent to which Anti-egalitarianism and descriptive Meritocracy predict differently or not the support for the early release of the Black prisoner remains an exploratory question. If there is to be such an intergroup differentiation due to the endorsement of such an hierarchy-enhancing ideology, it is more likely that descriptive Meritocracy will capture it, as it is more normative and less influenced by social desirability than Anti-egalitarianism.

One extra level of complexity was added to this part of the analysis. In effect we intended to test this second hypothesis as to the absolute judgment of the agreement of the release of the Black prisoner, but also in a comparative stance in which we capture the difference between the agreement with the release of the Black versus the White prisoner. This option to double the analysis with both dependent variables is explicit in the analysis plan section of the pre-registration. This option is based on the knowledge that intergroup judgments are inherently comparative (e.g. [61]). The simple derogation of the outgroup member is indeed not the most subtle nor common way to express prejudice. More often than not, people are tempted to turn to ingroup bias rather than outgroup derogation, making ingroup favoritism a key measure [62, 63].

## Materials and methods

### Design and participants

A power analysis using G*Power 3.1 set the target sample size to 200 to provide .80 statistical power to detect a small to medium effect size of ƒ = 0.10, using alpha level of .05. The convenience sample of online participants was collected with Qualtrics through social media and we used two lotteries of 25€ each (in gift certificates) as an incentive. Data termination rule was 200 + 25% of registered participants. Qualtrics had recorded 11 “participants” that were instead mis-records from the testing phase of the questionnaire and were, as such, eliminated. As previously defined in the pre-registration (see below), we excluded 37 participants for not reaching the end of the questionnaire. Also, in line with exclusion criteria defined in pre-registration, we excluded 22 participants for not self-categorizing as White/ Portuguese White/ European Ascend. These participants had identified as Black/ Black Portuguese/ African descent (seven), Asian/ Asian Portuguese/ Asian descent (one), Gipsy/ Gipsy Portuguese/ Roma/ Gipsy descent (one) and as other (13) (e.g., Mixed, Latin, Brown, etc.).

The final sample size was formed by 180 participants, of which 135 were female, with ages ranging from 18 to 60 years old (*M* = 30.56, *SD* = 10.33), and with a political orientation ranging from far-left to far-right (*M* = 3.63, *SD* = 1.10, on a 7-point scale, in which lower values pertained to far-left and higher values to far-right). Most were lay participants, with only 16 (8.9%) describing to have worked in the legal field.

The participants were randomly assigned to a 2 (Colour of Skin of the Prisoner: Black vs. White) X 2 (Type of Crime: Stereotypically Black vs. Stereotypically White) mixed design, whereby type of crime was manipulated between-participants and the colour of the skin of the prisoners as a within-participants factor. 86 participants were in the condition with the stereotypically Black crime (with both White and Black prisoners) and 94 were presented the criminal cases with the stereotypically White crime (with both White and Black prisoners).

### Procedure

This study was pre-registered (osf.io/gmdu6) prior to data collection at Open Science Framework with an embargo of six months and it was approved by the Ethics Committee of the Institute of Social Sciences of the University of Lisbon (ref: 2020/12).

The study was collected online through the research group page on Facebook. After reading and accepting the informed consent, the study started by unravelling a cover story surrounding the bill proposed by the national government, through which due to the COVID-19 Pandemic public health issues and containment strategy, prisoners could be released earlier from prison. To assure ecological validity, we remembered public information regarding this bill, namely that it included some particular conditions of inclusion (e.g., applied only to prisoners who have less than two years of sentence to serve) and some particular exclusions (e.g., not for prisoners who committed murder). We completed our cover story by accentuating the controversy surrounding the bill as grounds to investigate what is the opinion of the general public as to the earlier release from prison in concrete cases. Participants were then told they would see six criminal cases and asked to what extent they would agree with the early release from prison of each one. We continued by asking some questions on the “participant’s views of the world”, the section where we included the legitimizing beliefs scales. We concluded by asking sociodemographic variables. At the end, participants were debriefed and invited to apply for two lotteries of €25 in vouchers.

### Materials

#### Criminal cases

The cases consisted of mock Court Notifications and Judgments of the Supreme Judicial Court. Participants saw a total of 6 criminal cases, of which only two were critical. The others were irrelevant for the research and were kept constant. The critical cases were always the third and the sixth cases. Prisoners from these cases had approximately the same sociodemographic information (age, gender) and legal information, which was the basis of our manipulations, as explained below (type of crime committed, overall sentence time, time left to serve). The only variation was the skin colour of the prisoner. On the top of each Judgment of the Supreme Judicial Court, we included a photograph of the “prisoner” as a way of manipulating defendants’ racial category. These photographs were downloaded from the Face Research Lab London Set project [64]. Faces were blurred in order to exclude effects of facial features stereotypicality [22]. The order of the presentation was made stable, having always the Black defendant first, so to ensure that participants regarded this information at an intergroup level [65]. We built four critical criminal cases in total, two of them specifically for the White-stereotyped crime condition (embezzlement) and the other two for the Black stereotyped crime condition (car theft). The initial selection of the crimes was made following a large body of literature on racially stereotyped crimes [29, 66, 67], which was later confirmed by a pretest. Embezzlement was rated significantly higher in White stereotypicality (*M* = 5.61, *SD* = 1.22, 95% CI [5.29, 5.94]), than Car Theft (*M* = 3.96, *SD* = 1.44, 95% CI [3.56, 4.36]). The reverse pattern is described as to Black stereotypicality, with car theft (*M* = 5.33, *SD* = 1.48, 95% CI [4.92, 5.74]) higher in this dimension than embezzlement (*M* = 4.00, *SD* = 1.70, 95% CI [3.55, 4.45]).

#### Agreement with early release from prison due to COVID-19 pandemic

After describing each criminal case we asked the participants to rate to which degree they agreed with the early release from prison for each prisoner, in a scale ranging from: 1 = *Totally Disagree* to 7 = *Totally Agree*.

#### Descriptive Meritocracy scale

Participants completed a descriptive Meritocracy scale [60]. The scale comprised 15 items (sample items: “Success is possible for anyone who is willing to work hard”; “This is an open society in which everyone can attain a higher status”), that were answered from 1 = *Totally Disagree* to 7 = *Totally Agree*. An exploratory factor analysis revealed a one-factor solution explaining 48.91% and comprising 14 items (factor loadings from .554 to .803). One item had unsatisfactory loading (-.264) and was taken from the analysis. The Descriptive Meritocracy index was thus computed by averaging 14 items of the scale (*α* = .916, *M* = 3.89, *SD* = 1.10).

#### Anti-egalitarianism scale

We used three items of the pro-trait Anti-egalitarianism dimension (sample item: “Group equality should not be our primary goal”) from the SDO_7_ Scale [58]. By error a fourth item pertaining to the pro-trait dominance subdimension was included in the questionnaire. All analysis described in the paper were run with and without this fourth item, and given that no difference was found, results were reported with the index composed of only the three Anti-egalitarianism items. Participants were asked to rate their agreement with each item on a 7-point rating scale from 1 = *Strongly oppose* to 7 = *Strongly in favour*. An exploratory factor analysis revealed a one-factor solution explaining 69.35% of the variance and comprising all items (factor loadings from .737 to .881). An Anti-egalitarianism index was computed by averaging three items of the scale (*α* = .768, *M* = 2.14, *SD* = 1.39).

#### Sociodemographic information

In the end, participants answered to demographic items such as, sex, age, political orientation (ranging from 1 = *Far-Left* and 7 = *Far-Right*), their self-categorization into minority groups and if they worked in the legal field.

## Results

In line with the criteria defined in the pre-registration, we computed analyses without outliers of more than 2.5 standard deviations.

We start by noting that the overall agreement with early release of prisoners due to the COVID-19 pandemic was low for both the Black prisoners (*M* = 2.96, *SD* = 1.84) and the White prisoners (*M* = 3.03, *SD* = 1.86). In both cases the level of agreement with the release of the prisoners was below the midpoint of the agreement scale (*t*_*Black*_(179) = -7.618, *p* ≤ .001; *t*_*White*_(179) = -6.970, *p* ≤ .001, respectively).

Furthermore, the repeated measures ANOVA with Prisoner´s skin colour (Black vs. White) as a within factor and Stereotypicality of the Crime (Black vs. White) as a fixed factor revealed no main effect of the skin colour of the prisoner (*F*(1, 178) = 1.331, *p* = .250).

**Fig 1:**
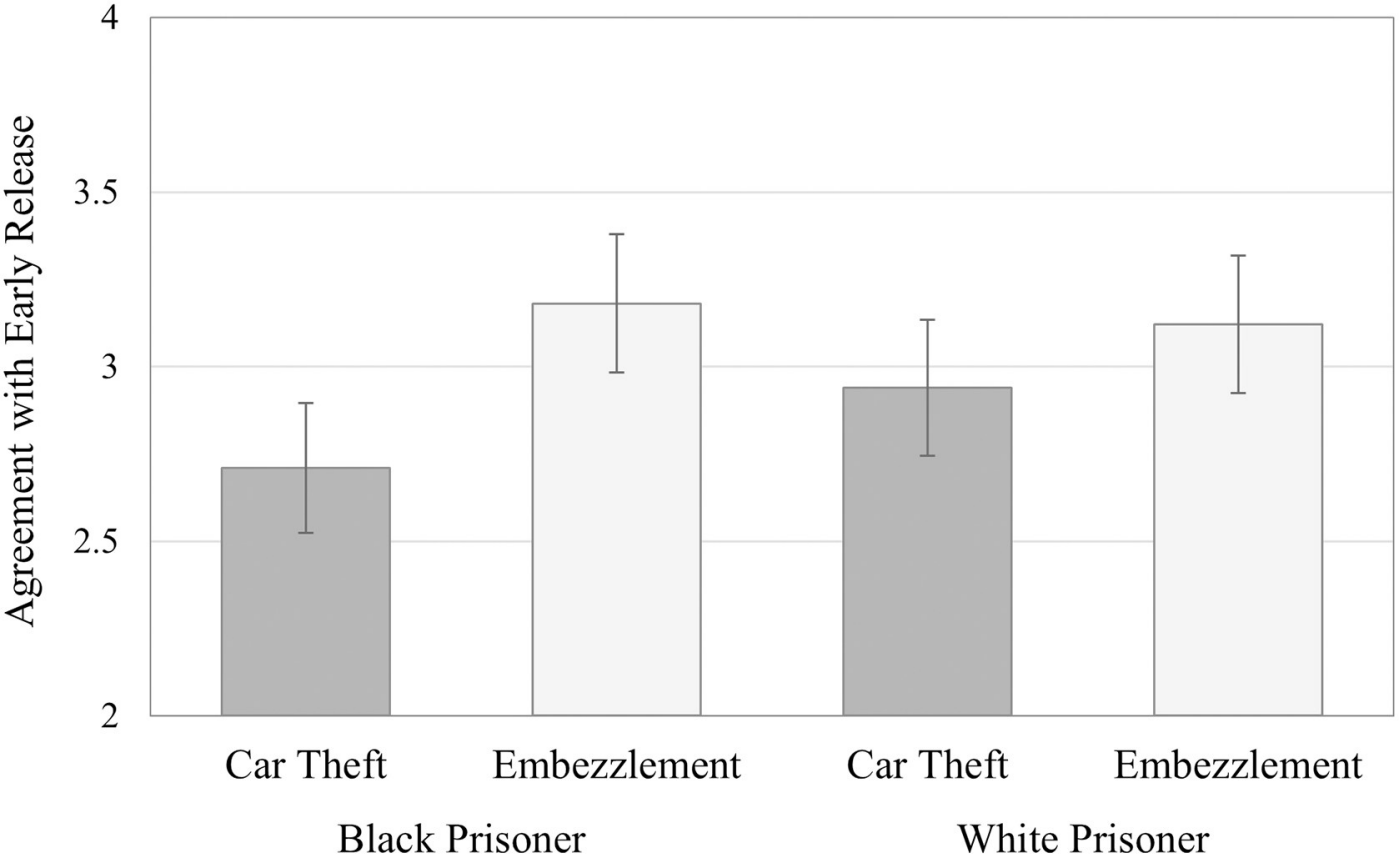
Bias in agreement with early release. Means (and standard errors) in levels of agreement with early release from prison due to COVID-19 pandemic, in function of the prisoner’s skin colour and of the type of crime committed.

Most importantly, and in line with our first hypothesis, there is a significant interaction between the prisoners’ skin colour and the stereotypicality of the crime (*F*(1, 178) = 4.108, *p* = .044, *η*_*p*_^*2*^ = .023). Contrast analysis, with Sidak adjustment, shows that this effect indeed corresponds to a significant lower agreement with the release of the Black prisoner (*M* = 2.71, *SD* = 1.73) compared to the White prisoner (*M* = 2.94, *SD* = 1.81), when both committed car theft, the stereotypically Black crime (*F*(1, 178) = 4.843, *p* = .029, *η*_*p*_^*2*^ = .026). No such difference appeared when the crime committed was embezzlement, the stereotypically White crime, with equal agreement towards the release of Black (*M* = 3.18, *SD* = 1.73) and White prisoners (*M* = 3.12, *SD* = 1.91, *F*(1, 178) = .399, *p* = .529) (v. Fig 1).

To test our second hypothesis, we included hierarchy-enhancing beliefs in the analyses. First, we started by running a hierarchical regression analysis focusing only on predicting the agreement with the release of the low-status target: the Black prisoner. In a first step, we included as predictors crime of stereotypicality, and the two hierarchy-enhancing beliefs. Results show type of crime was again a significant predictor, with lower agreement with the release of the Black prisoner when he had committed a stereotypically Black crime (*β* = -.169, *t*(164) = -2.283, *p* = .024). Moreover, there were significant main effects of both hierarchy-enhancing ideologies. The more the participants endorsed Meritocracy the less they agreed with the release of the Black prisoner (*β* = -.186, *t*(164) = -2.444, *p* = .016). The same pattern was found with Anti-egalitarianism, with higher levels of endorsement associated with less agreement with the release of the Black prisoner (*β* = -.179, *t*(164) = -2.364, *p* = .019). This model was in itself significant (*R*^*2*^ = .105, *F*(3, 165) = 6.458, *p* ≤ .001). We included a second level in the model to test if the effect of each of the hierarchy-enhancing ideologies on the opposition were particularly strong when the Black prisoner had committed a stereotypically Black crime. However, this second step did not present itself as a significant contribution to the model (*R*^*2*^ = .119, *F*_*change*_(2, 163) = 1.325, *p* = .269). This points to a cumulative, and not interactive, contribution of the cognitive and ideological factors. As such the first part of our second hypothesis is confirmed as the degree to which participants agree with the early release of Black prisoners are indeed negatively correlated to the endorsement of legitimizing beliefs, but the second part is not, as this effect is not particularly stronger for those who committed a stereotypically Black crime.

We then re-analysed the effect of the cognitive and ideological factors on the differences between groups, and as such we computed an index of the Intergroup Difference from the agreement with the early release of the Black prisoner minus the agreement with the early release of the White prisoner. Values close to zero indicate no intergroup difference, values above zero indicate more favourable decisions towards the Black prisoner, and negative values more favourable decisions towards the White prisoner.

We performed a hierarchical multiple regression with all the main variables under analysis: crime type, and the hierarchy enhancing endorsement as predictors of the Intergroup Differentiation Index. This full model was not significant (*R*^*2*^ = .051, *F*(5, 155) = 1.677, *p* = .143). However, we were more interested in the interaction terms, as these directly tested our second hypothesis that hierarchy-enhancing ideologies qualified the effect described in the first hypothesis: lower support for the Black prisoner, when the prisoner had been convicted of a stereotypically Black crime. When we added the interaction term between the Crime type and Anti-egalitarianism, we found that it does not improve the model (*R*^*2*^ = .025, *F*_*change*_(1, 156) = 1.019, *p* = .314). However, when we added, in a third step in the hierarchical model, the interaction between crime type and Meritocracy, we see that despite the model remaining non-significant, it indeed represents a significant improvement (*R*^*2*^ = .051, *F*_*change*_(1, 155) = 4.247, *p* = .041). In this overall model, all of the previously analysed predictors remained non-significant when explaining the difference between the support for the early release of the Black and the White targets—Meritocracy:*β* = -.042, *t*(154) = -.518, *p* = .605; Anti-egalitarianism: *β* = -.064, *t*(154) = -.788, *p* = .432; Crime Type: *β* = -.113, *t*(154) = -1.423, *p* = .157; the interaction between Anti-egalitarianism and Crime Type: *β* = .125, *t*(154) = 1.525, *p* = .129). The exception was therefore the significant effect of the interaction term between the endorsement of Meritocracy and the type of crime (*β* = -.168, *t*(154) = -2.061, *p* = .041).

**Fig 2:**
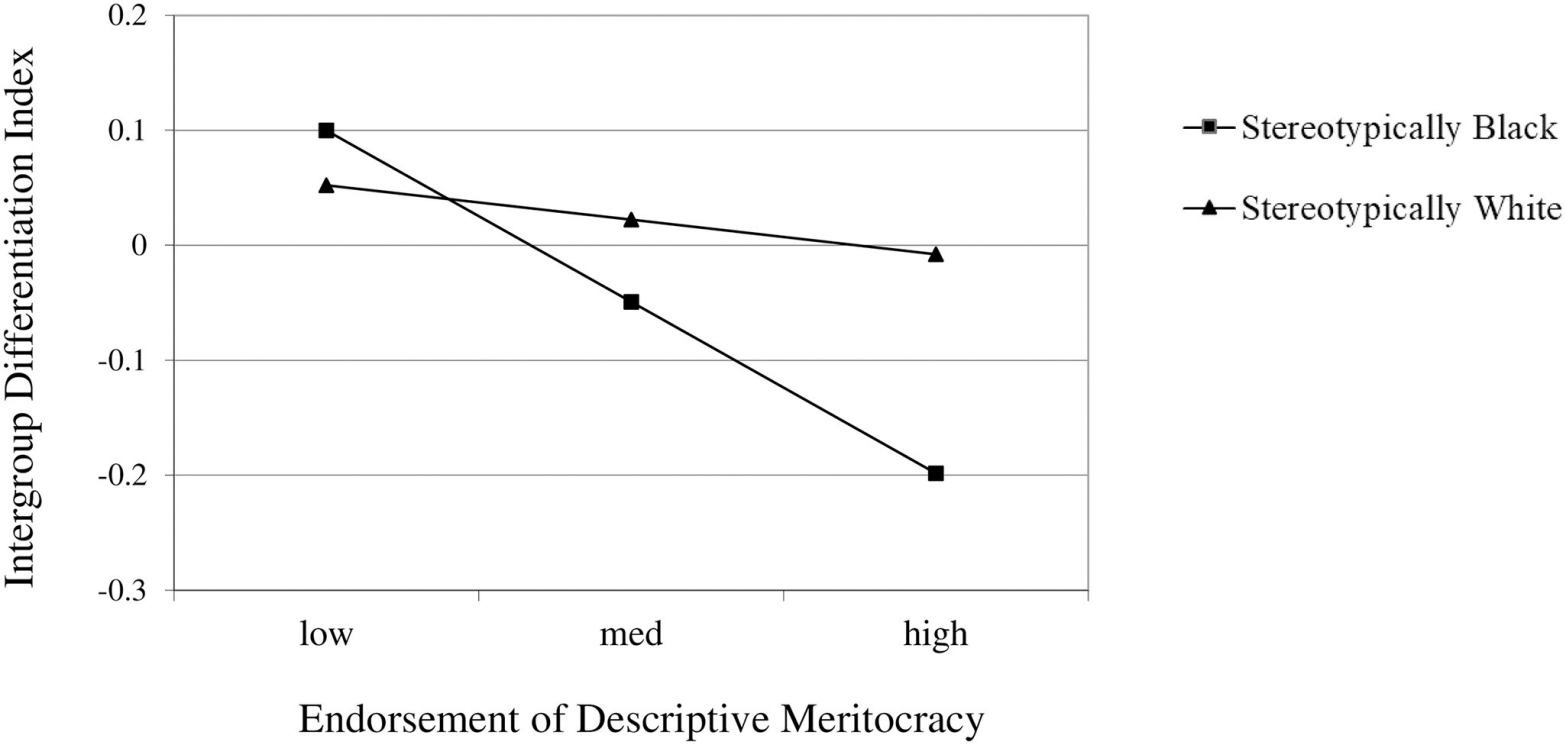
Stereotypes and Meritocracy interplay. Difference in the agreement with early release from prison between the Black and the White prisoners as a function of the interaction between the type of crime and levels of endorsement of descriptive Meritocracy.

Following recommendations for calculating simple slopes from the levels of the continuous variable at mean, mean plus and minus 1 standard deviation [68], we calculated the association between Meritocracy and the intergroup difference index for when the prisoners had committed a stereotypically Black or a stereotypically White crime (Fig 2).

Results showed that the interaction is due to a steeper slope in the association between higher levels of Meritocracy beliefs and unfavourable outcomes for the Black prisoner compared to the White prisoner when the crime committed was stereotypically Black (*b* = -.144, *SE* = .076, *p* = .061), than when it was stereotypically White (*b* = -.029, *SE* = .055, *p* = .597).

We continued by running a hierarchical regression analysis focusing only on the low-status target: the Black prisoner. We have done thusly so we could assert if the interaction effect of Type of Crime and Meritocracy that emerged as significant in predicting the difference in the support for early release is due to a specific change in the response to the Black target or is dependent of a relative judgment between the Black and White target. In a first step, we included the main effects model that yielded significance (*R*^*2*^ = .105, *F*(3, 165) = 6.458, *p* ≤ .001). Results showed that the interaction term added in a second step did not predict the agreement with the early release of the Black prisoner (*β* = -.041, *t*(163) = -.553, *p* = .581), making it a non-significant contribution for the model (*R*^*2*^ = .107, *F*_*change*_(1, 164) = .306, *p* = .581).

When we looked at the second hypothesis as to what is different in the agreement with the release of the Black prisoner with reference to what happens with the White prisoner is when we find a stance in which there is a complex integrative effect between crime stereotypicality and hierarchy-enhancing beliefs. However, our prediction is again only partially supported because there is a relative worse outcome for the Black prisoner who committed a stereotypically Black crime with higher levels of Meritocracy ideology, but not with Anti-Egalitarianism. In fact, in additional exploratory analyses, results show that, from all possible predictors, only the endorsement of anti-egalitarianism predicts (negatively) the agreement with early release of the White prisoner (see supplementary materials). This generalized effect of Anti-egalitarianism is discussed in the following section.

## Discussion

With this study we contribute to the understanding of the issue of racial disparities in the legal system. We do so by extending the research scope to the post-sentence phase. In particular, we show that the racial biases already detected in the four stages of the criminal path are also present in the early-release decisions, even when it represents an important measure to address the COVID-19 pandemic. In addition, we identify the factors underlying such biases by testing the predictive interplay of cognitive and ideological factors known to create and maintain these biases in the criminal system. The key empirical findings are twofold.

First, results clearly showed support for our first hypothesis, as participants differentiated between Black and White prisoners who committed a Black-stereotyped crime with equivalent legal situations by being less in favour of the early release of the Black prisoners. The solid meta-analytical evidence showing that crime stereotypicality plays a significant role in the sentencing phase [28] translated to this post-sentencing phase. This leap is of special importance when considering the impact of differential agency involved in decisions [69, 70]. In this context, the crime stereotypicality effect holds both in a situation where there is a more agentic posture (i.e., actively imprisoning a defendant) and a less agentic one (i.e., letting the prisoner stay in prison).

Second, the endorsement of hierarchy-enhancing legitimizing ideologies reinforced a general punitive tendency toward low-status group members, with more opposition to the early release of prisoners due to COVID-19. This was the case of participants who highly endorsed Anti-egalitarianism and descriptive Meritocracy. However, on the one hand, Anti-egalitarianism was a predictor of a general tendency for opposing early release of all prisoners, and not of an intergroup differentiation favouring the White prisoner. On the other hand, Meritocracy predicted a greater disadvantage for the Black prisoner, compared to the White, in the support for their early release.

As such, we only partially confirmed our second hypothesis, the complex interaction between cognitive and ideological factors appears only in what relates to Meritocracy, but not Anti-egalitarianism, and in the comparative judgment of the Black prisoner in relation to the White prisoner.

What our results seem to suggest is that participants with high levels of Meritocracy do not disadvantage Blacks blatantly, but instead favour Whites in comparison. This argument is especially plausible if we consider that all these judgments were made sequentially by participants and, as such, favouring a comparative stance. Moreover, it is of theoretical interest. Indeed, there is already evidence that White participants who are highly meritocratic seek to regard themselves as high in merit, and maintain this self-view by denying racial privilege. Also, this strategy of denying having benefitted from White privilege is preferred over anti-Black discrimination [71]. Other findings suggest that Whites’ concern for their own group and their concern for other minority groups can independently affect policy attitudes [72]. If we frame these results in an aversive racism approach [73], we can see that these highly-meritocratic participants do discriminate, but to do so have to make use of more subtle approaches, as favoring the ingroup more than derogating the outgroup and making use of stereotypes as a justification.

Meritocracy has been shown to be behind decisions that disfavour Black group members in many contexts [74]. For the first time, we show its association with bias in the context of the criminal system too. This effect is of particular importance considering that the way this experiment was designed and framed made the expression of prejudice more difficult. The task itself was totally explicit (i.e., same crime, one case following the other, explicit measure) and the setting of the cover-story was framed in relation to a need-base justice principle: the need to prevent overcrowding thus risking the widespread of the COVID-19 pandemic in the prison population. Nonetheless, Meritocracy maintains its relevance as a promoter of intergroup differentiation. We argue that it is exactly its apparent nature as equality provider that allows it to thrive.

One can argue that the fact that Anti-egalitarianism is indisputably opposed to the egalitarian norm may be the basis of the explanation of why we found a different pattern of results. However, two other alternative explanations should be examined. A first explanation relates to the possible difference in the effects of the two SDO subdimensions [58]. Here we have favoured the Anti-egalitarianism subdimension, that is related to more subtle hierarchy-enhancing legitimizing myths, in detriment of the Dominance subdimension. However, this second subdimension could capture the support for intergroup hostility and aggression, and agreement with domination and oppression of some groups by others, that can thrive in the kind of decisions involved in the criminal path. Examining the two subdimensions separately in future studies may allow us to predict, with more accuracy, intergroup attitudes and behaviors in the Criminal Justice System.

A second explanation also relates to context-specific information in this experiment. There is evidence that SDO does predict differences in sentencing decisions towards White and Black targets [75]. However, there is no research linking hierarchy-enhancing ideologies to post-sentencing decisions. Making decisions towards convicted criminals may have brought the Black and the White targets closer, as the White target is also a prisoner and thus is seen as having a very low-status independently of skin colour [57]. People high in SDO react poorly to social mobility from the low to high-status groups, opposing for instance assimilation strategies from minority group members [76] and also probably the reintegration of former prisoners. As such, future research should control for attributions of status for both groups and, therefore, test this explanation.

These alternatives to the different adaptation of hierarchy-enhancing ideologies to specific contexts underscore the need to include Meritocracy in the tradition of comparing the effects and motivation underlying different hierarchy-enhancing ideologies. We can see in the literature theoretical and empirical efforts to compare SDO and Right-Wing Authoritarianism [77] and SDO and the Belief in the Just World [78]. We have made the case that Meritocracy should be included in these efforts informing a more encompassing comprehension of how hierarchy-enhancing beliefs legitimize unequal intergroup relations and contributes to the reproduction of social inequality and the maintenance of systemic racism.

Finally, we wish to emphasize that not only was the endorsement of these ideologies related with the bias towards the Black prisoner, but a specific configuration occurred when the crime was stereotypically Black. In particular, the endorsement of descriptive Meritocracy predicts a greater difference in the opposition towards the Black compared to the White prisoner, only when they had committed a stereotypically Black crime. In this case, cognitive and ideological factors interact. One can argue that the apparently egalitarian norm of Meritocracy needs a justification for this differentiation between the Black and the White prisoners, and that the stereotypicality of the crime and the stability of behaviour that people infer from the stereotype [79] might be such justification.

Taken together, these results show the pervasiveness of stereotypical and ideological roots of bias found in previous research addressing preliminary phases of the legal path [13]. To the best of our knowledge, this study is the first direct comparison of factors from two levels of explanation [80]; a comparison that reveals a cumulative not competitive explanatory power of cognitive and ideological factors.

In spite of the contribution of our results, there are potential limitations that should be addressed. We stress two types: the first related to the sampling options and the second to the design options. We start by addressing the sample issues. We defined a specific sample size, with both a minimum sample size according to power analysis, and also a maximum sample size in line with the best practices of limiting p-hacking strategies (in particular, continuing to add participants until significance is reached, [81]). For this we set a goal of collecting an extra 25% of the minimum number of participants, but after applying all exclusion criteria the end sample was smaller than the established minimum. Also, with convenience sampling strategies there is always a concern with self-selection bias. This, in turn, should warn for caution in attempts of generalization of our results.

As to the design limitations, we highlight that this study did not allow for a complete control of order conditions. We presented the Black defendant first and the White defendant second to ensure that the intergroup frame of reference was activated [65]. That means that participants would focus their attention on intergroup differences and regard the information at an intergroup level. Also, by using a within-participants approach we make finding an effect according to our hypothesis more difficult, lowering the odds of a type 1 error. A second important limitation relates to the choice of the dependent variable. While having a dichotomous variable (release vs. not release) would have reinforced external validity in reference to the legal actors who support courts to make such decisions, we chose to have a rating scale because we did not ask the participants for a decision in itself but for “political” support for such decisions, which, realistically, is more nuanced. As such, there is room in future studies to compare racial discrimination with the prisoner’s Race as a between factor and to map the outcomes in terms of a decision, especially if conducting the studies with judges and their legal support teams. Finally, we acknowledge that this study did not focus on the people who are the actual decision-makers. Nonetheless, we find several accounts of responsiveness of policy makers to public opinion [82], with several accounts of policy gridlocks due to lack of public support [83].

The responsiveness effect has also been described in the context of the criminal justice system [84], in which we can observe a transition from a responsiveness logic (the democracy-at-work thesis) [85] to a populist one. This “penal populism” effect consists in the exploitation of punitive public sentiment for political gain, driving the implementation of the more punitive measures [86].

Most public policies researchers within the criminal justice system highlight public opinion as a complex, nuanced and changeable process. As such, they advise against research based on the average opinion poll results and argue for a deeper and more comprehensive understanding of the factors contributing to its development [85, 87]. Following the work in parallel fields that highlight individual social psychological variables as strong predictors of policy support [83], this work in the field of social and political psychology can serve to advance our understanding of the social and cultural construction of public opinion and political preferences in the criminal justice system.

When developing public policies that seek helping low status or minority group members within the criminal justice system, it is important to question whether these ad hoc policies do not merely constitute another circumstance where racial disparities are able to emerge. Here we have shown that, when crime stereotypicality is salient and the endorsement of Meritocracy as a hierarchy-enhancing belief is high, it just might.

## Supporting information

S1 File Analyses of the predictors for the White prisoner. (DOCX)

S2 File OSF pre-registration. (PDF)

S1 Data (SAV)
