# Supplementary material for: Early release from prison in time of COVID-19: Determinants of unfavourable decisions towards Black prisoners
Source: PLoS One. 2021 May 27;16(5):e0252319. doi: 10.1371/journal.pone.0252319 (PMC8158868; doi:10.1371/journal.pone.0252319)
Supplement: S1 File — (DOCX) [file pone.0252319.s001.docx]

**Agreement of Early Release of the White Prisoner**

We run an exploratory hierarchical regression analysis focusing only on the White prisoner. In a first step, we included the cognitive and hierarchy-enhancing ideologies as predictors of the early release of the White prisoner. This model was in itself significant (*R^2^* = .083, *F*(3, 165) = 4.962, *p* = .003). However, neither crime stereotypicality (*β* = -.135, *t*(164) = -.988, *p* = .325), nor Meritocracy (*β* = -.186, *t*(164) = -2.444, *p* = .016) were significant predictors. The only significant effect was of Anti-egalitarianism (*β* = -.390, *t*(164) = -3.036, *p* = .003), so that the more participants endorse Anti-egalitarianism the less they agree with the early release of the White prisoner. Results showed that the interaction term of antiegalitarianism and crime stereotypicality added in a second step did not make it a significant contribution for the model (*R^2^* = .085, *F_change_*(1, 164) = .504, *p* = .504), nor did adding a third the interaction term of Meritocracy and crime stereotypicality (*R^2^* = .085, *F_change_*(1, 163) = .002, *p* = .969) .

Table. Hierarchical Regression Analysis of Predictors of Support for the Early Release of the White Prisoner

|  | **Model 1** | | | **Model 2** | | | **Model 3** | | |
| --- | --- | --- | --- | --- | --- | --- | --- | --- | --- |
| *Variable* | *b* | *SE* | *β* | *b* | *SE* | *β* | *b* | *SE* | *β* |
| Crime Stereotypicality | -.135 | .137 | -.074 | -.121 | .139 | -.066 | -.121 | .139 | -.066 |
| Anti-Egalitarianism | -.390 | .129 | -.233* | -.401 | .130 | -.239* | -.401 | .130 | -.239* |
| Meritocracy | -.197 | .135 | -.112 | -.196 | .136 | -.111 | -.196 | .136 | -.111 |
| Anti-Egalitarianism* Crime Stereotypicality |  |  |  | .085 | .126 | .051 | .083 | .130 | .050 |
| Meritocracy* Crime Stereotypicality |  |  |  |  |  |  | .005 | .136 | .003 |
| R^2^ | .083 | | | .085 | | | .085 | | |
| Fchange in R^2^ | 4.962* | | | .449 | | | .002 | | |

Note: * p-value < .01
