# Supplementary material for: Early release from prison in time of COVID-19: Determinants of unfavourable decisions towards Black prisoners
Source: PLoS One. 2021 May 27;16(5):e0252319. doi: 10.1371/journal.pone.0252319 (PMC8158868; doi:10.1371/journal.pone.0252319)
Supplement: S2 File — (PDF) [file pone.0252319.s002.pdf]

SCD_LEGAL

A. Hypotheses - Essential elements

Description of essential elements

Describe the (numbered) hypotheses in terms of directional relationships between your (manipulated or measured) variables.

H1) Participants will disagree more with the early release of Black prisoners than their White counterparts when the crime committed is stereotypically Black.
H2) We also expect that the degree to which participants agree with early release of Black prisoners will be negatively correlated to the endorsement of legitimizing beliefs, in particular for those who committed a stereotypically Black crime.

For interaction effects, describe the expected shape of the interactions.

H1) We expect that participants will disagree more with early release of Black prisoners than White prisoners when crime is stereotypically Black. We do not predict the same effect in White prisoners comparing to Black prisoners when they commit a stereotypically White crime.

If you are manipulating a variable, make predictions for successful check variables or explain why no manipulation check is included.

We are not including a manipulation check of the color of skin of the participants, because it is a self-evident manipulation. As to the stereotypicality of the crime, it is a manipulation based on a large body of literature on racially stereotyped crimes (Boetcher, 2009; Jones & Kaplan, 2003; Skorinko & Spellman, 2013; Sunnafrank & Fontes, 1983).

Recommended elements

Recommended elements

A figure or table may be helpful to describe complex interactions; this facilitates correct specification of the ordering of all group means.

No files selected

For original research, add rationales or theoretical frameworks for why a certain hypothesis is tested.

No response

If multiple predictions can be made for the same IV-DV combination, describe what outcome would be predicted by which theory.

No response

B. Methods - Essential elements

Description of essential elements

Design

List, based on your hypotheses from section A:

Independent variables with all their levels
  a. whether they are within- or between-participant
  b. the relationship between them (e.g., orthogonal, nested).

2 (Target: White vs. Black) X 2(Crime stereotypicality: White vs. Black), whereby crime stereotypicality will be manipulated between-subjects and the target within-subjects.

List dependent variables, or variables in a correlational design

Agreement with early release from prison due to COVID-19 Pandemic.

Third variables acting as covariates or moderators.

Descriptive meritocracy scale.
Anti-Egalitarianism scale.

Planned Sample

If applicable, describe pre-selection rules.

N/A

Indicate where, from whom and how the data will be collected.

The survey will be distributed online, using a convenience sample.

Justify planned sample size

We will aim at a sample of a minimum of 200 participants to achieve a power of .80, with $\alpha = 0.05$ and an expected effect size of $ƒ = 0.10$.

If applicable, you can upload a file related to your power analysis here (e.g., a protocol of power analyses from G*Power, a script, a screenshot, etc.).

No files selected

Describe data collection termination rule.

Data will be terminated when we reach 200+25% participants, for a total of 250 participants.

Exclusion Criteria

Describe anticipated specific data exclusion criteria. For example:

  a) missing, erroneous, or overly consistent responses;

b) failing check-tests or suspicion probes;
c) demographic exclusions;
d) data-based outlier criteria;
e) method-based outlier criteria (e.g. too short or long response times).

a) Missing - We will exclude the participants who do not reach the end of the dependent variables.
c) Demographic - Participants who do not self-categorize as White/ Portuguese White/ European Ascend
d) Outlier - We will conduct outlier analysis and exclude data that deviates more than 2.5 standard deviations from the mean of each variable (comparable analysis with and without outliers will be run and reported on footnotes).

Procedure

Describe all manipulations, measures, materials and procedures including the order of presentation and the method of randomization and blinding (e.g., single or double blind), as in a published Methods section.

After reading and accepting the informed consent, we will start by explaining that due to the COVID-19 Pandemic the government is proposing a bill in which prisoners can be released earlier from prison. We will say that that bill was made in a way that it included some particular conditions of inclusion (e.g. applied only to prisoners who have less than two years of sentence to serve) and some particular exclusions (e.g. not for prisoners who committed murder). However, due to much controversy surronding the law, it is important to find out what the public thinks.
They will be told they will see 6 criminal cases and asked to what degree they agree with the early release from prison of each one.
We will continue by asking some questions on the “participant's views of the world” (section where we will include the legitimizing beliefs: the Meritocracy Scale; Anti-egalitarianism scale). We will end with sociodemographic variables.
Finally, participants will be debriefed and asked to include their email in a different link to be able to apply for two lotteries of €25 in vouchers.

- Criminal cases. We built eight criminal cases, four of them mutual to both conditions and irrelevant for this research, two of them specifically for the White-stereotyped crime condition and the other two for the Black-stereotyped crime condition: theft will be the Black-stereotyped crime and embezzlement the one used as a White-stereotyped crime. The cases consisted of mock Legal/ Court Notifications and Judgments of the Supreme Judicial Court, based on real ones. For each crime condition only two criminal cases out of the six cases presented were of interest to this research, namely the third and the sixth cases (Black and White defendants, respectively). The order of the presentation was made stable, having always the Black defendant first, so to ensure that participants regarded this information at an intergroup level (Simon, 1995). Defendants from these cases had the same age, gender and committed crime. On the top of each Judgment of the Supreme Judicial Court, we included a photograph of the “prisoner” as a way of manipulating defendants’ racial category. These photographs were downloaded from the Face Research Lab London Set project (DeBruine & Jones, 2017). Faces were blurred in order to exclude effects of facial features stereotypicality (Eberhardt et al., 2004).
- Dependent Variable. After describing the criminal case, sentence time and sentence still left, we will ask the participants to what degree do they agree with the early release from prison for each prisoner, ranging from: 1 = Totally Disagree to 7 = Totally Agree
- Meritocracy Scale. A scale of 15 items of descriptive meritocracy (Madeira, 2019), ranging from 1 = Totally Disagree to 7 = Totally Agree.
- Egalitarianism Scale (SDO-E; Ho et al., 2015) – we will use the 4 items of anti-egalitarianism (e.g., “Group equality should not be our primary goal”). Participants are asked to rate their agreement with each item on 7-point rating scale from 1 = “Strongly oppose” to 7 = “Strongly in favor”. The scale was validated to Portugal (Carvalho et al., 2020).
- Sociodemographic information. Then, participants will answer to demographic items of relevance for this work, sex, age, political orientation (ranging from 1 = Far-Left and 7 = Far-Right), their self-categorization into ethnic groups and if they work in the legal field.

Recommended elements

Recommended elements

Procedure

Set fail-safe levels of exclusion at which the whole study needs to be stopped, altered, and restarted. You may pre-determine what proportion of excluded participants will cause the study to be stopped and restarted.

No response

If applicable, you can upload any files related to your methods and procedure here (e.g., a paper describing a scale you are using, experimenter instructions, etc.)

No files selected

C. Analysis plan - Essential elements

Confirmatory Analyses

Describe the analyses that will test the first main prediction from the hypotheses section. Include:

the relevant variables and how they are calculated;

DV: Agreement with early release
IV1: Crime stereotypicality (Stereotypically Black vs Stereotypically White) (between factor)
IV2: Target (White vs Black prisoner) (within factor)
None Of the variables result from transformed or computed information.

the statistical technique;

We will calculate a mixed ANOVA, with all the design included (2x2)
Using SPSS – Analysis - General Linear Model – Repeated Measures – Test of Within Subjects-Effect
We will run contrasts with sidak adjustments to compare Black and White prisoners when they have commited the stereotypically white Crime and the Black and White prisoners when they have commited stereotypically Black crime.

each variable's role in the technique (e.g., IV, DV, moderator, mediator, covariate);

DV: Agreement with early release
IV1: Crime stereotypicality (Stereotypically Black vs Stereotypically White) (between factor)
IV2: Target (White vs Black prisoner) (within factor)

rationale for each covariate used, if any;

N/A

if using techniques other than null hypothesis testing (for example, Bayesian statistics), describe your criteria and inputs toward making an evidential conclusion, including prior values or distributions.

N/A

Second Prediction

Describe the analyses that will test the second main prediction from the hypotheses section. Include:

the relevant variables and how they are calculated;

the relevant variables and how they are calculated;

DVa: Agreement with early release for Black prisoner
[or DVb: Difference in agreement with early between Black and White targets (Computed through the difference in the agreement with early release for Black prisoner and the agreement with early release from the White prisoner)]

IV1: Crime stereotypicality (Stereotypicality Black vs Stereotypicality White) (dummy transformed -1 and 1)

IV2: Legitimizing beliefs [IV2a) Meritocracy index OR IV2b) Anti-egalitarianism index]
[Both this scales are going to be analysed in an exploratory factor analysis and reliability analysis – According to that we will compute one or several indices of each construct, averaging the items that saturated in each factor. All of these new variables will be mean-centered]

IV1*IV2 – Interaction between crime stereotypical_dummy and IV2A_mean centered OR IV2B_mean centered (computed by multiplying both variables after they have been transformed into a dummy variable or mean-centered, respectively)

the statistical technique;

Using SPSS – Analysis - Regression – Linear
The expected interaction will be analysed through an excel macro by Jose (2002)

each variable's role in the technique (e.g., IV, DV, moderator, mediator, covariate);

DV: Agreement with early release for Black prisoner [and a second independent analysis for DVB: Difference in agreement with early between Black and White targets]

IV1: Crime stereotypicality_Dummy

IV2: Legitimizing beliefs_Mean Centered [IV2a) Meritocracy index and a second analysis with IV2b) Anti-egalitarianism index]

IV1*IV2 – Interaction between crime stereotypical and legitimizing beliefs [IV2a) Meritocracy index and a second analysis with IV2b) Anti-egalitarianism index]

rationale for each covariate used, if any;

N/A

if using techniques other than null hypothesis testing (for example, Bayesian statistics), describe your criteria and inputs toward making an evidential conclusion, including prior values or distributions.

N/A

Third Prediction

Describe the analyses that will test the third main prediction from the hypotheses section. Include:

the relevant variables and how they are calculated;

No response

the statistical technique;

No response

each variable's role in the technique (e.g., IV, DV, moderator, mediator, covariate);

No response

rationale for each covariate used, if any;

No response

if using techniques other than null hypothesis testing (for example, Bayesian statistics), describe your criteria and inputs toward making an evidential conclusion, including prior values or distributions.

No response

Fourth Prediction

Describe the analyses that will test the fourth main prediction from the hypotheses section. Include:

the relevant variables and how they are calculated;

No response

the statistical technique;

No response

each variable's role in the technique (e.g., IV, DV, moderator, mediator, covariate);

No response

rationale for each covariate used, if any;

No response

if using techniques other than null hypothesis testing (for example, Bayesian statistics), describe your criteria and inputs toward making an evidential conclusion, including prior values or distributions.

No response

Further Predictions

Describe the analyses that will test any further (main) predictions from the hypotheses section. Include:

the relevant variables and how they are calculated;

No response

the statistical technique;

No response

each variable's role in the technique (e.g., IV, DV, moderator, mediator, covariate);

No response

rationale for each covariate used, if any;

No response

if using techniques other than null hypothesis testing (for example, Bayesian statistics), describe your criteria and inputs toward making an evidential conclusion, including prior values or distributions.

No response

[illegible]

Recommended elements

Recommended Elements

Specify contingencies and assumptions, such as:

Method of correction for multiple tests.

No response

The method of missing data handling (e.g., pairwise or listwise deletion, imputation, interpolation).

No response

Reliability criteria for item inclusion in scale.

No response

Anticipated data transformations.

No response

Assumptions of analyses, and plans for alternative/corrected analyses if each assumption is violated.

No response

Optionally, upload any files here that are related to your analyses (e.g., syntaxes, scripts, etc.).

No files selected

Final questions

Has data collection begun for this project?

No, data collection has not begun

If data collection has begun, have you looked at the data?

No

The (estimated) start and end dates for this project are

23.04.2020 - Starting date
23.10.2020 - Ending date

Any additional comments before I pre-register this project

No response

Copyright © 2011-2020 Center for Open Science (https://cos.io) | Terms of Use (https://github.com/CenterForOpenScience/centerforopenscience.org/blob/master/TERMS_OF_USE.md) | Privacy Policy (https://github.com/CenterForOpenScience/centerforopenscience.org/blob/master/PRIVACY_POLICY.md) | Status (https://status.cos.io/) | API (https://developer.osf.io/)
TOP Guidelines (http://cos.io/top/) | Reproducibility Project: Psychology (https://osf.io/ezcuj/wiki/home/) | Reproducibility Project: Cancer Biology (https://osf.io/e81xl/wiki/home/)

(http://twitter.com/OSFramework) (https://www.facebook.com/CenterForOpenScience/)
